# Long‐term stability evaluation of user‐derived electrometer calibration factors (*k*_elec, user_) for radiotherapy dosimetry

**DOI:** 10.1002/acm2.70840

**Published:** 2026-10-08

**Authors:** Hayato Tsuno, Koji Sasaki, Ruan Sasaki, Fumiyasu Matsubayashi, Kohei Nishi, Tae Ushikawa, Daisaku Goto, Ririko Kosugi, Yasuhiro Kawashima

**Affiliations:** ^1^ Gunma Prefectural College of Health Sciences Maebashi Gunma JAPAN; ^2^ Department of Radiation Oncology Japanese Red Cross Maebashi Hospital Maebashi Gunma JAPAN; ^3^ Graduate School of Radiological Technology Gunma Prefectural College of Health Sciences Maebashi Gunma JAPAN; ^4^ Department of Radiology National Hospital Organization Sendai Medical Center Sendai Miyagi JAPAN; ^5^ Department of Radiation Oncology Cancer Institute Hospital, Japanese Foundation for Cancer Research Tokyo JAPAN; ^6^ Department of Radiology JR Tokyo General Hospital Tokyo JAPAN; ^7^ Department of Radiology Hamamatsu University School of Medicine Hamamatsu Shizuoka JAPAN; ^8^ Department of Medical Technology Japanese Red Cross Maebashi Hospital Maebashi Gunma JAPAN

**Keywords:** electrometer, electrometer calibration, long‐term stability, quality assurance, radiotherapy dosimetry

## Abstract

**Purpose:**

User‐derived electrometer calibration factors (*k*_elec, user_) have been proposed as a practical approach for electrometer quality assurance in radiotherapy dosimetry. However, the contribution of long‐term stability to the uncertainty of *k*_elec, user_ has not been quantitatively evaluated. This study aimed to assess the long‐term stability of *k*_elec, user_ over a 28‐month period and to validate the method using different signal input techniques.

**Methods:**

A RAMTEC Solo electrometer was used as the reference electrometer, and three electrometers (UNIDOS^webline^, RAMTEC Duo, and Super MAX) were evaluated as electrometers under test. Electrometer sensitivity ratios were measured using three signal input methods: A LINAC‐based method, a battery‐powered current source (SCG002), and a servo‐controlled current source (EMF521R). User‐derived calibration factors were calculated from the sensitivity ratios and the calibration coefficient of the reference electrometer. Long‐term stability was evaluated from repeated measurements over 28 months using linear regression analysis, and the associated uncertainty component was estimated according to the Guide to the Expression of Uncertainty in Measurement (GUM).

**Results:**

The coefficients of variation for repeated measurements were ≤ 0.071% for all methods. The *k*_elec, user_ values obtained using the LINAC method, SCG002, and EMF521R agreed within 0.1%, with a maximum relative difference of 0.06%. Over the 28‐month observation period, the largest estimated drift was 1.84 × 10^−^^4^, corresponding to a Type B standard uncertainty of 1.06 × 10^−^^4^ (1.06 × 10^−^^2^%). The combined standard uncertainty of *k*_elec, user_ remained 0.20%, and the experimentally determined long‐term stability component had a negligible effect on the overall uncertainty.

**Conclusions:**

The influence of signal input method on *k*_elec, user_ was minimal, and consistent results were obtained using LINAC‐based and current‐source‐based measurements. The uncertainty contribution from long‐term stability over 28 months was negligible compared with the reported uncertainty of electrometer calibration factors. These findings support the use of periodic in‐house sensitivity comparison measurements as a complementary quality assurance tool for monitoring electrometer performance between external calibrations. These findings extend previous studies by providing quantitative evidence of the relative sensitivity stability of user‐derived electrometer calibration factors and thereby support their application for routine in‐house quality assurance.

## 1 INTRODUCTION

Reference dosimetry in external beam radiotherapy is performed using ionization chambers and electrometers to determine absorbed dose to water.^1^, ^2^, ^3^ Electrometers are essential measurement devices that quantify the charge generated in ionization chambers, and their response characteristics directly affect the accuracy of absorbed dose determination. Calibration laboratories provide each electrometer with an electrometer calibration coefficient, (*k*_elec_), which is used to correct the measured charge.

Appropriate quality assurance (QA) is required to maintain electrometer performance, and the importance of standalone electrometer performance evaluation and periodic stability verification has been reported.^4^, ^5^ Methods for evaluating electrometer response by applying known charges or currents directly to the electrometer, without using a medical linear accelerator (LINAC), have also been proposed and shown to be useful. ^6^, ^7^

Recently, it has been reported that sensitivity coefficients for an electrometer can be derived from the calibration information of a reference electrometer when adequate QA is performed and stable sensitivity ratios are maintained among electrometers.^8^, ^9^, ^10^ This approach may enable the sharing of calibration information among multiple electrometers, improve the efficiency of electrometer QA, and potentially allow the use of a user‐derived calibration coefficient as an alternative to assuming (*k*_elec _= 1.0000).^10^

However, long‐term application of such user‐derived electrometer sensitivity coefficients requires quantitative evaluation of temporal changes in sensitivity ratios and determination of the uncertainty associated with long‐term stability. In addition, the characteristics of the current source used for electrometer evaluation may influence measurement results depending on its operating principle.^8^, ^9^, ^10^ In particular, current sources based on resistive components may exhibit changes in effective output current due to the input resistance of the connected electrometer, according to Ohm's law.^10^ Nevertheless, previous studies have indicated that the input resistance of electrometers generally remains stable unless the instrument is modified or repaired.^10^ Therefore, evaluating the stability of electrometer sensitivity ratios using signal input methods based on different operating principles is important for validating the reliability of the measurement results.

In recent years, the concept of in‐situ calibration, in which instruments are calibrated without being removed from the measurement system, has gained increasing attention in the field of metrology.^11^, ^12^, ^13^ In radiotherapy dosimetry, if the long‐term stability of electrometer sensitivity can be demonstrated, continuous in‐house monitoring of electrometer performance may contribute to more efficient QA and potentially support future improvements in calibration practices. Therefore, the purpose of this study was to continuously measure electrometer sensitivity ratios over a period of up to 28 months, evaluate the uncertainty contribution associated with the long‐term stability of the user‐derived electrometer calibration coefficient (*k*_elec, user_), and investigate its potential usefulness for electrometer QA.

## 2 MATERIALS AND METHODS

### 2.1 Electrometers and calibration coefficients

The overall study design is illustrated in Figure 1. Four clinical electrometers were evaluated in this study: UNIDOS^webline^ (PTW, Freiburg, Germany), RAMTEC Duo and RAMTEC Solo (Toyo Medic Co., Ltd., Japan), and Super MAX (Standard Imaging, Middleton, WI, USA). All electrometers had calibration coefficients, *k*_elec_, assigned by an accredited calibration laboratory with traceability to the national standard. Electrometer calibration is performed independently of ionization chamber calibration, and each electrometer is assigned its own calibration coefficient.^3^, ^4^ None of the electrometers underwent external recalibration during the 28‐month observation period. Therefore, the calibration coefficient assigned to the reference electrometer (*k*_elec_) was treated as constant throughout the study period.

**FIGURE 1:**
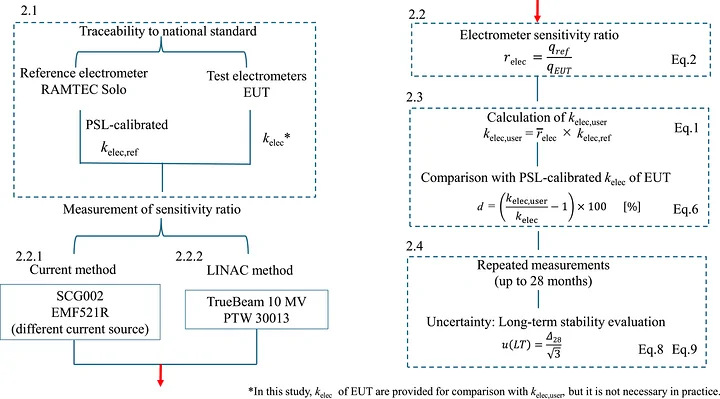
Schematic diagram of the study design. A traceable reference electrometer was used to determine the user‐derived calibration factor (*k*_elec, user_) of electrometers under test (EUTs). Electrometer sensitivity ratios were measured using the Current method and the LINAC method. Long‐term stability was evaluated from repeated measurements over a period of up to 28 months.

RAMTEC Solo was used as the reference electrometer, and the remaining electrometers were treated as electrometers under test (EUTs). The electrometer sensitivity ratio, ${\bar{r}}_{\mathrm{elec}}$, was determined from measurements obtained with the reference electrometer and each EUT. The user‐derived electrometer calibration coefficient, *k*_elec, user_, was then calculated as follows: ^8^, ^9^, ^10^

(1) $$k_{\mathrm{elec},\mathrm{user}}={\bar{r}}_{\mathrm{elec}}\;\times k_{\mathrm{elec},\mathrm{ref}}$$

where ${\bar{r}}_{\mathrm{elec}}$ is the mean sensitivity ratio obtained at three calibration points: Q_min_ (minimum charge), Q_0.5_ (50% of the maximum charge), and Q_max_ (maximum charge), and *k*_elec, ref_ is the calibration coefficient assigned to the reference electrometer.

### 2.2 Measurement of electrometer sensitivity ratio

Electrometer sensitivity ratios were measured using both the Current method and the LINAC method.^8^ The sensitivity ratio, $r_{\mathrm{elec}}$, was calculated as

(2) $$r_{\mathrm{elec}}=\frac{q_{\mathrm{ref}}}{q_{\mathrm{EUT}}}\;$$

where *q*_ref_ and *q*_EUT_ are the charge readings measured by the reference electrometer and the EUT, respectively, under identical charge input conditions. The measurement procedures were based on our previously reported electrometer cross‐calibration method,^8^, ^9^, ^10^ with the addition of long‐term follow‐up measurements and evaluation using two different current‐source systems.

Five repeated measurements were performed for each measurement condition to evaluate measurement reproducibility. The coefficient of variation (CV) and Type A standard uncertainty were calculated according to the Guide to the Expression of Uncertainty in Measurement (GUM).^14^ The CV was calculated as:

(3) $$CV=\frac{s}{\bar{x}}\;\times 100\;\left[\%\right]$$

where is the experimental standard deviation of repeated measurements and is the mean charge value.

The Type A standard uncertainty was calculated as the standard deviation of the mean:

(4) $$u_{A}=\frac{s}{\sqrt{n}}\;$$

where is the number of repeated measurements. For reporting in the Results section, the relative Type A standard uncertainty was calculated as:

(5) $$u_{\text{A,rel}}=\frac{u_{A}}{\bar{x}}\times 100=\frac{CV}{\sqrt{n}}\;\left[\%\right]$$

Therefore, all Type A uncertainty values reported in this study represent relative standard uncertainties normalized to the corresponding measured charge values.

#### 2.2.1 Current method

In this study, the electrometer sensitivity calibration procedure using electrical current sources was referred to as the Current method. Two current‐source devices were used as charge input sources: SCG002 (Kawaguchi Electric Works Co., Ltd., Japan) and EMF521R (EMF Japan Co., Ltd., Japan).

The use of current sources for electrometer performance evaluation has previously been reported as a practical method for assessing electrometer performance without using a medical linear accelerator.^6^, ^10^ The SCG002 is a battery‐powered current source that delivers charge over a predefined time interval controlled by an internal timer.^6^, ^10^ In this study, the SCG002 was operated for 60 s, and the average output current was calculated from the measured charge output.^10^ The irradiation time required to achieve the desired charge input was subsequently determined from the calculated current. The current sources were used as repeatable charge input devices rather than as absolute calibration standards.

The EMF521R is a servo‐controlled current source capable of generating a specified current output. Unlike the SCG002, the delivered charge can be directly controlled using the preset current and application time. The current magnitude was adjusted to match the average current determined using the SCG002.

Input charge levels were selected according to the measurement ranges of the electrometers and included a minimum charge (*Q*_min_), a maximum charge (*Q*_max_), and an intermediate charge corresponding to 50% of the maximum charge (*Q*_0.5_).^10^ The reference electrometer was measured first, followed by the EUT under identical conditions. Sensitivity ratios were then calculated from the measured charge values.

#### 2.2.2 LINAC method

For the LINAC method, a TrueBeam linear accelerator (Varian Medical Systems, Palo Alto, CA, USA) operated with a 10 MV photon beam was used. A Farmer‐type ionization chamber was positioned in a water phantom under reference conditions.^3^ Collected charge measurements were acquired using both the reference electrometer and each EUT under identical irradiation conditions.

Although electrometer intercomparison using a LINAC may be influenced by ionization chamber characteristics and accelerator output stability, it has been reported as a practical approach for electrometer comparison under clinical conditions.^7^ Measurement conditions and calculation procedures followed previously published methods.^5^

For each electrometer, irradiation conditions corresponding to *Q*_min_, *Q*_0.5_, and *Q*_max_ were established. In accordance with previous studies,^10^ *Q*_min_, *Q*_0.5_, and *Q*_max_ were set to 1 nC, 100 nC, and 200 nC, respectively. Sensitivity ratios were calculated from the charge readings obtained with the reference electrometer and each EUT. All measurements were performed under controlled environmental conditions. Electrometers were stored in a desiccator when not in use. During each measurement session, environmental conditions were maintained at approximately 25 ± 1.5°C and 50 ± 10% relative humidity. However, continuous recording of temperature and humidity over the entire 28‐month observation period was not available, and therefore detailed environmental variations could not be included in the present analysis.

### 2.3 Calculation and comparison of *k*_elec, user_

The *k*_elec, user_ values for each EUT were calculated using the sensitivity ratios obtained from the Current and LINAC methods.^8^ The mean sensitivity ratio measured at the three charge levels *Q*_min_, *Q*_0.5_, and *Q*_max_ was used for the calculation. The charge levels were 1 nC, 100 nC, and 200 nC, respectively.

The calculated *k*_elec, user_ values were compared with the traceable calibration coefficients *k*_elec_ assigned to each EUT. The relative difference, (*d*), was calculated as

(6) $$d=\left(\frac{k_{\text{elec, user}}}{k_{\text{elec}}}-1\right)\times 100\;\left[\%\right]$$

where (*d*) represents the percentage difference between the user‐derived calibration coefficient and the calibration coefficient assigned by the accredited calibration laboratory. In this study, the traceable *k*_elec_ values assigned to each EUT were treated as reference values, and the long‐term agreement between periodically determined *k*_elec, user_ values and the corresponding *k*_elec_ values was evaluated.

### 2.4 Long‐term stability evaluation

To evaluate the long‐term stability of electrometer sensitivity ratios, repeated measurements were performed over a period of 28 months. Temporal variations in *k*_elec, user_ obtained using each measurement method were plotted as a function of elapsed time. Measurements were performed periodically during the observation period according to available measurement opportunities in the clinical setting. Although the intervals were not uniform, the long‐term trend was evaluated using regression analysis over the entire observation period.

Long‐term temporal trends were evaluated using ordinary least‐squares linear regression. For each electrometer, a regression line was fitted with *k*_elec, user_ as the dependent variable and time of measurement (*t*) as the independent variable. The regression slope (*b*), its standard error (SE_b_), and 95% confidence interval (CI) were calculated. Because nine measurement time points were available for each electrometer, the degrees of freedom for the slope estimate were *n* − 2 = 7, where two degrees of freedom are consumed by estimating the slope and intercept of the linear regression. The 95% CI for the slope was calculated as:

(7) $$95\%\;CI=b\;\pm \;t_{0.975,7}\;\times \;SE_{b}$$

where *t*_0.975,7_ is the critical value of the Student's *t*‐distribution with 7 degrees of freedom for a two‐sided 95% CI. The drift magnitude (*Δ*) over the observation period (*T*) was estimated from the absolute value of the regression slope as:

(8) Δ=∣b∣T

The uncertainty associated with long‐term stability was evaluated as a Type B uncertainty according to the Guide to the Expression of Uncertainty in Measurement (GUM).^14^ Because neither the timing nor the direction of the drift could be predicted a priori, the drift was assumed to follow a rectangular distribution over the observation period. The standard uncertainty associated with long‐term drift, *u*(*LT*), was therefore calculated as

(9) $$u\left(\mathrm{LT}\right)=\frac{\Delta_{28}}{\sqrt{3}}$$

where (*Δ*_28 _= $|b|T_{28}$) is the estimated drift over the 28‐month observation period derived from the regression analysis. The term represents the long‐term stability uncertainty of either the reference electrometer or the electrometer under test (EUT).

For each electrometer, the uncertainty associated with long‐term stability was evaluated from the observed temporal variation using Equation (9). The sensitivity ratio was determined relative to RAMTEC Solo; therefore, the observed temporal variation represents the combined effect of possible changes in the reference electrometer and the electrometer under evaluation. Accordingly, the present study does not independently separate the long‐term drift of the reference electrometer from that of the EUT. Instead, the long‐term stability of each electrometer was characterized by the temporal variation of the measured sensitivity ratio over the 28‐month observation period. For the uncertainty budget, the largest long‐term stability uncertainty obtained among the evaluated electrometers was adopted as a conservative worst‐case estimate.

## 3 RESULTS

### 3.1 Reproducibility assessment

Table 1 summarizes the reproducibility results for each charge input method. The worst‐case values among the evaluated electrometers are presented. All measurement methods showed low variability, with coefficients of variation below 0.1%, indicating sufficient reproducibility for electrometer sensitivity comparison measurements.

**TABLE 1:** Repeatability of charge measurements obtained using the TrueBeam LINAC, EMF521R, and SCG002. Measurements were performed with the RAMTEC Solo as the reference electrometer in this study. The mean collected charge, coefficient of variation (CV), and Type A standard uncertainty are presented from five repeated measurements.

Current Source	TrueBeam	EMF521R	SCG002
AVE [nC]	16.11	16.0617	16.0522
SD [nC]	1.1 × 10^−2^	6.1 × 10^−4^	1.4 ×10^−4^
CV [%]	7.1 × 10^−2^	3.8 × 10^−3^	8.9 × 10^−4^
Uncertainty type A [%]	2.2 × 10^−2^	1.2 × 10^−3^	2.8 × 10^−4^

Abbreviations: AVE: average; CV: coefficient of variation; SD: standard deviation.

### 3.2 Results of *k*_elec, user_

Table 2 summarizes the sensitivity ratios and calculated *k*_elec, user_ values obtained using the LINAC and Current methods. The values obtained using the different signal input methods showed good agreement for all evaluated electrometers.

**TABLE 2:** Electrometer sensitivity ratios (*r*_elec_), user‐derived electrometer calibration factors (*k*_elec, user_), calibration coefficients (*k*_elec_), and relative errors (*d*) obtained using the TrueBeam LINAC, EMF521R, and SCG002 for each electrometer under test.

	Electrometer								
	UNIDOS^webline^			RAMTEC Duo			SuperMAX		
	Linac method	Current method		Linac method	Current method		Linac method	Current method	
Parameter	TrueBeam	SCG002	EMF521R	TrueBeam	SCG002	EMF521R	TrueBeam	SCG002	EMF521R
	0.9999	—	—	1.0009	—	—	0.9991	—	—
	—	0.9992	0.9993	—	1.0002	1.0011	—	0.9989	0.9987
*k*_elec, ref_	1.0001	1.0001	1.0001						
*k*_elec, user_	1.0000	0.9993	0.9994	1.0010	1.0003	1.0012	0.9992	0.9990	0.9988
*k*_elec_	0.9998	1.0006	0.9986						
*d* [%]	0.020	−0.050	−0.040	0.040	−0.030	0.060	0.060	0.040	0.020

The maximum difference between the LINAC and Current methods was 0.06%, and all calculated *k*_elec, user_ values agreed within 0.1%. The relative difference (*d*) between *k*_elec, user_ and the traceable calibration coefficient *k*_elec_ was within 0.06% for all electrometers, which was smaller than the previously reported expanded uncertainty of *k*_elec, user_ (0.20%).^9^

### 3.3 Long‐term stability of *k*_elec, user_

Figure 2 shows the long‐term stability of *k*_elec, user_ over the 28‐month observation period. The horizontal axis represents elapsed time (months), and the vertical axis represents *k*_elec, user_. The sensitivity ratios were determined relative to RAMTEC Solo, which was used as the reference electrometer throughout the study. Linear regression equations obtained using the least‐squares method are also shown.

**FIGURE 2:**
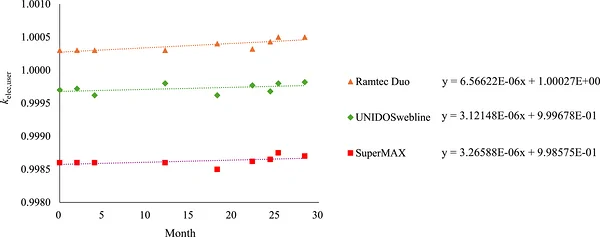
Long‐term stability of the user‐derived electrometer calibration factor (*k*_elec, user_) over 28 months, evaluated using the SCG002 as the signal‐input device. The horizontal axis represents elapsed time in months and the vertical axis represents *k*_elec, user_. Symbols indicate individual electrometers under test, and dotted lines represent linear regression fits used for drift estimation.

Table 3 summarizes the regression slopes, standard errors (SEs), and 95% confidence intervals (CIs) for the three evaluated electrometers. The regression slopes were positive for all three electrometers. The slope was 6.566 × 10^−6^ month^−1^ for RAMTEC Duo, 3.121 × 10^−6^ month^−1^ for UNIDOS^webline^, and 3.266 × 10^−6^ month^−1^ for Super MAX. The 95% CI for the slope did not include zero for RAMTEC Duo, whereas the CIs for UNIDOS^webline^ and Super MAX included zero.

**TABLE 3:** The slope, standard error (SE) of the slope, and 95% confidence interval (CI) were obtained by ordinary least‐squares linear regression over the 28‐month observation period. The sensitivity ratios were determined relative to RAMTEC Solo, which was used as the reference electrometer.

Electrometer	Slope (/month)	SE of slope (/month)	95% CIs of slope (/month)
RAMTEC Duo	6.566 × 10^−6^	1.686 × 10^−6^	2.58 × 10^−6^ ～ 1.06 × 10^−5^
UNIDOS^webline^	3.121 × 10^−6^	2.371 × 10^−6^	−2.48 × 10^−6^ ～ 8.73 × 10^−6^
Super MAX	3.266 × 10^−6^	2.108 × 10^−6^	−1.72 × 10^−6^ ～ 8.25 × 10^−6^

For RAMTEC Duo, which showed the largest absolute regression slope, the estimated drift over the 28‐month observation period was 1.86 × 10^−4^ (Equation 8). Assuming a rectangular distribution, the corresponding Type B standard uncertainty associated with long‐term stability was 1.08 × 10^−4^% (Equation 9). This value was adopted as the conservative long‐term stability component in the uncertainty budget.

### 3.4 Uncertainty budget analysis

Table 4 summarizes the uncertainty budget for the determination of *k*_elec, user_ and compares the uncertainty components used in the previous study with those obtained in the present study. The combined standard uncertainty remained 0.20%, and the corresponding expanded uncertainty (*k* = 2) remained 0.40%.

**TABLE 4:** Uncertainty budget for the determination of *k*_elec, user_. Relative standard uncertainties obtained in the present study are compared with previously reported values.

	Relative standard uncertainty				
Performance characteristic	Tsuno et al^10^		This study		Remarks/assumptions relating standard to conditions of measurement
Display resolution	0.0029	%	0.0029	%	The display resolution varies depending on the electrometer's specifications, but to avoid underestimating the uncertainty, half the maximum display resolution of 0.01 nC was used as the representative value. The sensitivity coefficient was calculated as the coefficient of variation when the sensitivity was changed by 0.01 nC.
Long‐term‐stability *u*(LT)	0.058 ^4^	%	0.011(Equation 9)	%	1.5 years after calibration 4
Zero shift	0.058	%	0.058	%	Percentage of signal charge ^4^
Stability time	0.17	%	0.17	%	After 1 h response should be half way to final value ^4^
Temp. response	0.0075	%	0.0075	%	Used within 5°C of calibration temperature ^4^
*q*_ref_	0.0019	%	0.032	%	The worst‐case coefficient of variation was used as the limit. Measurements were repeated five times, and the estimate was made using Type A.
*q*_EUT_	0.0021	%	0.032	%	The worst‐case coefficient of variation was used as the limit. Measurements were repeated five times, and the estimate was made using Type A.
*k*_elec_	0.080	%	0.080	%	The calibration certificate uncertainty (*k *= 1) was used as the representative value.
*u*_c_	0.20	%	0.20	%	
*U *(*k =* 2)	0.40	%	0.40	%	

*u*_c_:Combined standard uncertainty

*U*:Expanded uncertainty

The largest uncertainty components were the stabilization time (0.17%), electrometer calibration coefficient *k*_elec_ (0.08%), and zero shift (0.058%). In contrast, the uncertainty component attributed to long‐term stability was 1.1 × 10^−4^%, which was substantially smaller than the value of 0.058% assumed in the previous study. Thus, the experimentally determined long‐term stability component was approximately one‐sixth of the previously assumed value.

Although repeated measurements contributed to the Type A uncertainty, the uncertainty components associated with charge measurements *q*_ref_ and *q*_EUT_ remained small relative to the dominant Type B components. Consequently, incorporation of the experimentally determined long‐term stability component had a negligible effect on the overall uncertainty budget, and the combined standard uncertainty remained unchanged at 0.20%.

## 4 DISCUSSION

In this study, electrometer sensitivity ratios were measured using different charge input methods, and their long‐term stability was evaluated over a period of up to 28 months. First, the reproducibility of the measurement system and the applicability of current‐source‐based measurements are discussed, followed by consideration of the long‐term stability of *k*_elec, user_ and its clinical significance.

Tsuno et al. evaluated repeated measurements of collected charge using multiple linear accelerators and reported that a coefficient of variation (CV) below 0.10% is required for electrometer comparison measurements using a LINAC.^15^, ^16^ In the present study, the maximum CV observed in charge measurements was 0.071%, which was comparable to the previously reported values. These results indicate that the charge input methods and measurement procedures used in this study provided sufficient reproducibility for evaluating electrometer sensitivity ratios.

Next, the electrometer evaluation method using current sources is discussed. The LINAC‐based electrometer comparison method has the advantage of utilizing clinically relevant irradiation conditions; however, it requires dedicated LINAC time and coordination with patient treatments and other QA activities. Furthermore, operational constraints may arise when a large number of electrometers must be evaluated continuously within a facility.^8^

In contrast, the Current method enables verification of electrometer sensitivity changes without using a LINAC and therefore has potential applications in institutional quality control programs.^10^ In this study, two current sources based on different operating principles—a battery‐powered current source (SCG002) and a servo‐controlled current source (EMF521R)—were compared, and the resulting electrometer sensitivity ratios showed good agreement.

The current output of EMF521R was intentionally matched to that of SCG002 to enable a controlled comparison between two current sources with different operating principles. The SCG002 may be influenced by the effective resistance of the connected electrometer system because its output current is determined by a resistive circuit, whereas the EMF521R maintains the preset current using a servo‐control mechanism. Therefore, the agreement between these two systems suggests that the influence of current‐source operating characteristics on electrometer sensitivity‐ratio measurements was limited under the investigated conditions.

In addition, the current output of EMF521R was evaluated under conditions adjusted to approximate LINAC‐generated signals, and no meaningful difference in sensitivity ratios was observed. The agreement among the LINAC method, SCG002, and EMF521R indicates that the influence of signal input characteristics on electrometer sensitivity‐ratio measurements was limited under the investigated conditions.

However, when applying current‐source‐based measurements to electrometer quality control, the characteristics of both the current source and the electrometer should be considered. In particular, the output characteristics of a battery‐powered current source may be affected by the input impedance and internal circuitry of the electrometer, including the guard electrode configuration.^10^ Therefore, verification of current‐source stability and confirmation that the electrometer input circuitry has not been altered by repair or modification remain important. Within the range of electrometers and measurement conditions investigated in this study, the results support the use of current‐source‐based sensitivity‐ratio measurements as a practical complementary approach to LINAC‐based intercomparison.

Having discussed the reproducibility and validity of the measurement methods, the impact of long‐term stability on the uncertainty of *k*_elec, user_ is considered. Tsuno et al. reported an uncertainty of 0.20% (*k* = 1) for *k*_elec, user_, including contributions from long‐term stability.^10^ In the present study, the temporal trends in *k*_elec, user_ were evaluated using ordinary least‐squares linear regression. The regression slopes were positive for all three evaluated electrometers. The slope for RAMTEC Duo was 6.566 × 10^−^^6^ per month, whereas those for UNIDOS^webline^ and Super MAX were 3.121 × 10^−^^6^ and 3.266 × 10^−^^6^ per month, respectively. The 95% confidence interval (CI) for the slope did not include zero for RAMTEC Duo, whereas the CIs for UNIDOS^webline^ and Super MAX included zero. Thus, a positive temporal trend was observed for all three electrometers, although the statistical evidence for a non‐zero trend differed among the electrometers.

The standard uncertainty attributable to long‐term stability was estimated from the largest observed regression slope. For RAMTEC Duo, the estimated drift over the 28‐month observation period was 1.84 × 10^−^^4^, corresponding to a Type B standard uncertainty of 1.06 × 10^−^^4^, or 1.06 × 10^−^^2^%. According to the Guide to the Expression of Uncertainty in Measurement (GUM), uncertainty components that are sufficiently small compared with dominant components may be neglected in practice.^14^ Furthermore, when an uncertainty component is less than one‐tenth of the largest contributor, its effect on the combined standard uncertainty becomes negligible after root‐sum‐of‐squares combination.^10^

The uncertainty attributable to long‐term stability obtained in this study was substantially smaller than the major uncertainty components contributing to *k*_elec, user_ and therefore had a limited impact on the expanded uncertainty. As shown in Table 4, the standard uncertainty associated with long‐term stability was 1.06 × 10^−^^2^%, which was considerably smaller than those associated with the calibration coefficient *k*_elec_ (0.080%), stabilization time (0.17%), and zero shift (0.058%). Castro et al. reported that the calibration coefficient *k*_elec_ contains an uncertainty of approximately 0.25% (*k* = 1).^17^ Compared with these values, the uncertainty due to long‐term stability observed in this study was several orders of magnitude smaller, indicating that long‐term stability is not a major contributor to the uncertainty of electrometer calibration factors when appropriate quality control is maintained.

The regression slope and its 95% CI characterize the statistical uncertainty of the observed temporal trend, whereas the Type B uncertainty assigned to long‐term stability represents an uncertainty allowance for potential future variation. Because the magnitude and direction of future drift cannot be predicted a priori, the latter was evaluated using a rectangular distribution. However, it should be noted that the stability evaluated in this study represents relative stability rather than absolute accuracy. External calibration ensures traceability to national standards and establishes the absolute value of *k*_elec_. In contrast, institutional sensitivity comparisons are intended to monitor temporal changes through relative comparison with a reference electrometer.

The calculated *k*_elec, user_ values exhibited high stability throughout the 28‐month observation period, and the contribution of long‐term stability to the overall uncertainty was negligible. In addition, both the certified *k*_elec_ values and the calculated *k*_elec, user_ values for the electrometers investigated in this study were close to unity. These findings suggest that, for institutional quality control and condition monitoring purposes, the possibility of approximating *k*_elec_ as 1.0000 may warrant further investigation. However, the present study did not evaluate the absolute accuracy of electrometer calibration coefficients and does not replace traceability to national standards. Therefore, additional studies are required to define the appropriate scope of such an approach.

Having discussed the reproducibility and long‐term stability, the clinical significance of the proposed sensitivity‐comparison method is considered. External calibration ensures traceability to national standards and establishes the absolute value of *k*_elec_. In contrast, institutional sensitivity comparisons are intended to monitor drift and abnormal changes through relative comparison with a reference electrometer. Therefore, these approaches should be regarded as complementary rather than interchangeable quality‐control methods.

The findings of this study do not eliminate the need for external calibration; rather, they support the use of periodic in‐house measurements as a supplementary method for monitoring electrometer performance between calibration intervals. Routine sensitivity‐ratio measurements may facilitate early detection of abnormal sensitivity changes or impending failures, thereby improving the reliability of dosimetry systems used in radiotherapy.

Furthermore, this study does not replace previously proposed electrometer sensitivity‐comparison methods. Instead, it provides additional evidence regarding their long‐term applicability by demonstrating that sensitivity ratios remain stable over a 28‐month period and are consistent among multiple signal input methods based on different operating principles. Earlier studies demonstrated short‐term feasibility and reproducibility, whereas the present study quantified long‐term stability over a period of up to 28 months. The agreement observed among multiple signal input methods based on different operating principles further supports the validity of the previously proposed methodology.

Therefore, the proposed method should be considered a complementary QA tool rather than a replacement for conventional electrometer intercomparison. Its potential role is to provide additional opportunities for verifying electrometer sensitivity stability between external calibrations, particularly in situations where frequent LINAC‐based measurements are impractical.

In recent years, the concept of in‐situ calibration, in which instruments are calibrated or verified without removal from the measurement system, has been increasingly investigated in fields such as building engineering and industrial metrology.^11^, ^12^, ^13^ In radiotherapy, sending electrometers or dosimeters away for external calibration may affect the scheduling of patient‐specific QA and LINAC QA activities. The electrometer sensitivity‐ratio evaluation approach presented in this study enables periodic monitoring of electrometer performance and may provide foundational evidence for future in‐house quality‐control programs and the development of in‐situ verification strategies in radiotherapy. The potential benefit of the proposed approach is not an increase in measurement accuracy beyond conventional intercomparison methods, but rather improved flexibility in implementing routine electrometer monitoring. Because current‐source‐based measurements do not require LINAC operation, they may facilitate periodic verification of electrometer performance without interfering with clinical activities.

Several limitations should be acknowledged. First, the number and types of electrometers evaluated were limited, and further studies are required before the findings can be generalized to all electrometer models. Second, the Current method assumes that the electrometer input circuitry and guard‐electrode configuration remain unchanged; therefore, re‐evaluation may be necessary following repair or hardware modification. Third, because the sensitivity ratios were determined relative to RAMTEC Solo, which served as the reference electrometer throughout the study, the observed temporal variation represents the combined effect of possible changes in the reference electrometer and the electrometer under evaluation. The present study therefore cannot independently separate the long‐term drift of the reference electrometer from that of the EUT. An independent traceable reference system would be required to distinguish these contributions. Finally, the present study evaluated long‐term stability over a period of 28 months, and this observation period does not represent the entire clinical lifetime of electrometers, which may exceed 10 years. Further long‐term investigations involving larger datasets and extended observation periods are required to fully characterize electrometer stability over its clinical lifetime.

## 5 CONCLUSION

In this study, electrometer sensitivity ratios were measured repeatedly over a period of up to 28 months, and the uncertainty associated with the long‐term stability of the user‐derived electrometer calibration factor (*k*_elec, user_) was evaluated. Comparisons among different signal input methods (LINAC method, Current method using the SCG002, and Current method using the EMF521R) showed good agreement, indicating that the influence of the input method on the measured sensitivity ratio was minimal.

The uncertainty associated with long‐term stability was estimated to be 1.06 × 10^−^^2^% in the conservative worst‐case estimate based on the largest observed temporal trend, which was substantially smaller than the reported uncertainty of *k*_elec, user_. These findings suggest that, for electrometers maintained under appropriate quality management programs, long‐term stability is not a major contributor to the uncertainty of *k*_elec, user_.

The present study extends the validation of a previously proposed electrometer intercomparison method by demonstrating its relative sensitivity stability over a 28‐month observation period and across multiple signal input techniques. Although the proposed approach does not replace external calibration and traceability to national standards, periodic in‐house electrometer sensitivity verification may provide a practical means of detecting long‐term drift and abnormal behavior. The findings may also serve as a basis for future investigations of complementary in‐house quality management strategies and in‐situ verification concepts in radiotherapy dosimetry.

## AUTHOR CONTRIBUTIONS

Hayato Tsuno conceived and designed the study and drafted the manuscript with support from Koji Sasaki. Hayato Tsuno, Ruan Sasaki, and Fumiyasu Matsubayashi performed the data analysis and interpretation. Koji Sasaki, Fumiyasu Matsubayashi, Ruan Sasaki, Kohei Nishi, Tae Ushikawa, Daisaku Goto, and Yasuhiro Kawashima contributed to the study design and critically revised the manuscript. Ririko Kosugi contributed to the interpretation of the results and critically reviewed the manuscript. All authors read and approved the final manuscript.

## CONFLICT OF INTEREST STATEMENT

There are no conflicts of interest to declare.

## ETHICS STATEMENT

This study was a technical investigation involving electrometer measurements and did not involve human participants, human tissue, or identifiable personal data. Therefore, ethical review was not required

## Data Availability

Authors will share data upon request to the corresponding author.
